# Phylogenetic Analysis Shows That Neolithic Slate Plaques from the Southwestern Iberian Peninsula Are Not Genealogical Recording Systems

**DOI:** 10.1371/journal.pone.0088296

**Published:** 2014-02-18

**Authors:** Daniel García Rivero, Michael J. O'Brien

**Affiliations:** 1 Department of Prehistory and Archaeology, University of Seville, Seville, Spain; 2 Department of Anthropology, University of Missouri, Columbia, Missouri, United States of America; New York State Museum, United States of America

## Abstract

Prehistoric material culture proposed to be symbolic in nature has been the object of considerable archaeological work from diverse theoretical perspectives, yet rarely are methodological tools used to test the interpretations. The lack of testing is often justified by invoking the opinion that the slippery nature of past human symbolism cannot easily be tackled by the scientific method. One such case, from the southwestern Iberian Peninsula, involves engraved stone plaques from megalithic funerary monuments dating ca. 3,500–2,750 _B.C._ (calibrated age). One widely accepted proposal is that the plaques are ancient mnemonic devices that record genealogies. The analysis reported here demonstrates that this is not the case, even when the most supportive data and techniques are used. Rather, we suspect there was a common ideological background to the use of plaques that overlay the southwestern Iberian Peninsula, with little or no geographic patterning. This would entail a cultural system in which plaque design was based on a fundamental core idea, with a number of mutable and variable elements surrounding it.

## Introduction

**Figure 1:**
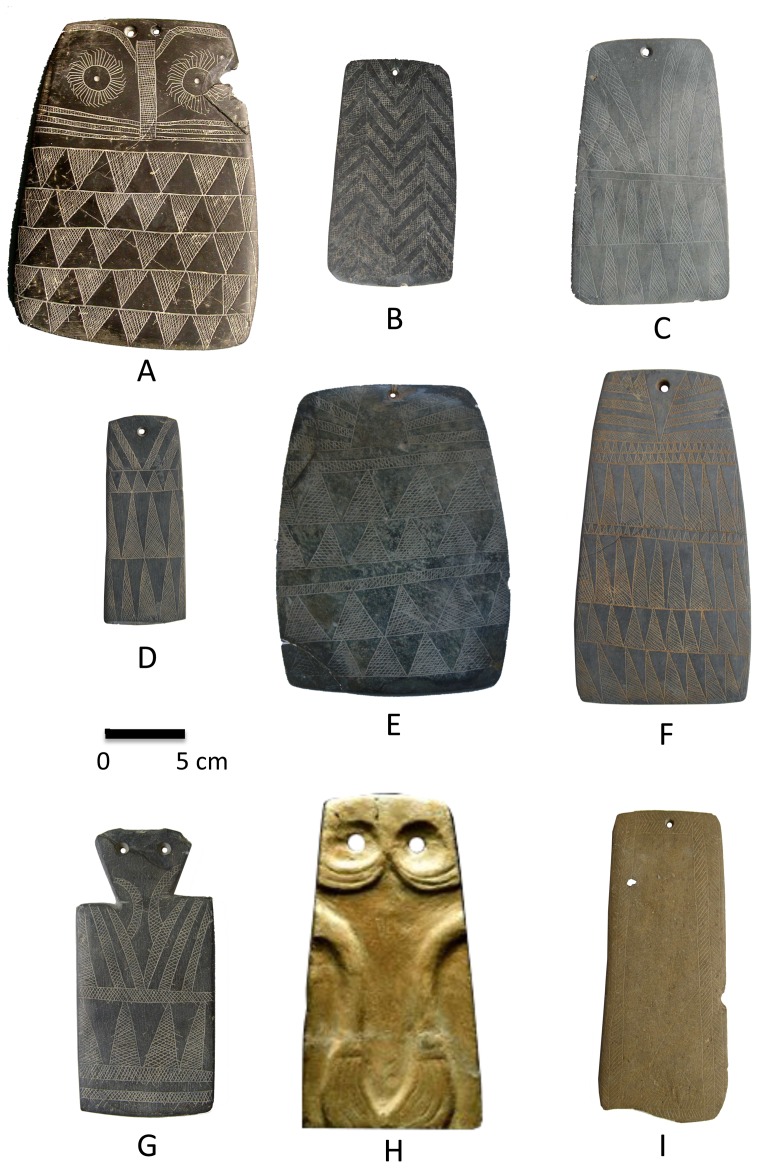
Engraved plaques from the Iberian Peninsula. a, Valencina de la Concepción, Sevilla, Spain (Museo Arqueológico de Sevilla [MAS]); b, S. Geraldo, Montemor-o-Novo, Évora, Portugal (Museo Nacional de Arqueologia de Portugal [MNAP]); c, Monsaraz, Reguengos de Monsaraz, Évora (MNAP); d, Mora, Évora (MNAP); e, Jabugo, Aracena, Huelva, Spain (MAS); f, Ciborro, Monte-o-Novo, Évora (MNAP); g, Marvão, Portalegre, Portugal (MNAP); h, Estremoz, Évora (MNAP); and I, Pavia, Mora, Évora (MNAP).

Prehistoric engraved plaques dating ca. 3,500–2,750 _B.C._ (calibrated age) (Table 1) are found in archaeological sites across the southwestern Iberian Peninsula. The plaques are thin slabs, usually of slate or schist but in some cases of sandstone, that vary in shape from rectangular to trapezoidal. Size is highly variable, but most specimens range in length from 10 cm to 20 cm and have maximum widths in the 5–10-cm range (Fig. 1). In addition to geometric, anthropomorphic, or zoomorphic designs engraved on one face, most specimens have one or two drilled holes at one end, through which, it has been proposed [1], [2], strings were passed so they could be worn. The majority of plaques have come from burial sites (∼200), in some cases resting directly on or to the side of human skeletons [2]. Plaques are usually associated with undecorated pottery, flint blades, and other chipped and polished stone tools with no clear evidence of wear [2].

**Table 1:** Available radiocarbon dates directly associated with plaques.

Site and District^1^	Sample	Date RCYBP	Date BC	Cal Date BC^2^(1 sigma)	Plaque (Esprit Number)	Reference
Gruta da Lapa do Fumo (Set)	ICEN-240	4420±45 BP	2470±45 BC	3101–3000 BC	658	[82]
Covas das Lapas I (Lei)	ICEN-463	4550±60 BP	2600±60 BC	3238–3108 BC	1103	[20]
Gruta 2 da Marmota (San)	OxA-5535	4605±55 BP	2655±55 BC	3509–3426 BC	Unknown	[82]
Gruta da Lapa do Bugio (Set)	OxA-5507	4420±110 BP	2470±110 BC	3119–2919 BC	Unknown	[82]
Anta da Bola da Cera (Port)	ICEN-66	4360±50 BP	2410±50 BC	3023–2909 BC	Unknown	[81]
Sala n° 1 (Bej)	ICEN-448	4140±110 BP	2190±110 BC	2876–2618 BC	Unknown	[82]
Anta de STAM-3 (Evo)	Beta-166422	4270±40 BP	2320±40 BC	2917–2877 BC	650	[82]
Olival da Pega 2b (Evo)	ICEN-957	4130±60 BP	2180±60 BC	2763–2620 BC	137, 492, and 515	[82]
Olival da Pega 2b (Evo)	ICEN-955	4290±100 BP	2340±100 BC	3034–2856 BC	137, 492, and 515	[82]
Olival da Pega 2b (Evo)	ICEN-956	4180±80 BP	2230±80 BC	2817–2664 BC	137, 492, and 515	[82]
Anta 4 de Coureleiros (Port)	ICEN-976	4240±150 BP	2290±150 BC	3022–2617 BC	Unknown	[81]
Pé da Erra (San)	ICEN-587	4220±45 BP	2270±45 BC	2808–2755 BC	Unknown	[82]
Anta da Horta (Port)	Beta-194313	4480±40 BP	2530±40 BC	3332–3214 BC	[81] Figs. 138 (above) and 147 (below)	[81]
Anta da Horta (Port)	Beta-194312	4270±50 BP	2320±50 BC	2928–2866 BC	[81] Figs. 136–149	[81]
Gruta Praia das Maçãs (Lis)	OxA-5509	4410±75 BP	2460±75 BC	3107–2916 BC	Unknown	[82]
Gruta Praia das Maçãs (Lis)	OxA-5510	4395±60 BP	2445±60 BC	3096–2916 BC	Unknown	[82]

1 See Fig. 4 for district locations.

2 Calibrations are made in software Calib 7.0 based on IntCal 13 data sets.

Interpretations of the intended function(s) of the stone plaques extend back to the last quarter of the nineteenth century and include

- a kind of ideographic writing system [3];
- prestige objects [1], [4], [5];
- symbolic items used by groups within a social hierarchy [6], [7];
- heraldic objects [8], [9];
- amulets or cult objects [10], [11], perhaps used in superstitious activities [12];
- apotropaic images of the deceased to ward off evil [13];
- idols [14]–[16], perhaps related to the devotion of specific divine figures [17]–[21]; and
- symbolic expressions related to different specific geographical regions and units of cultural identity [22], [23].

**Figure 2:**
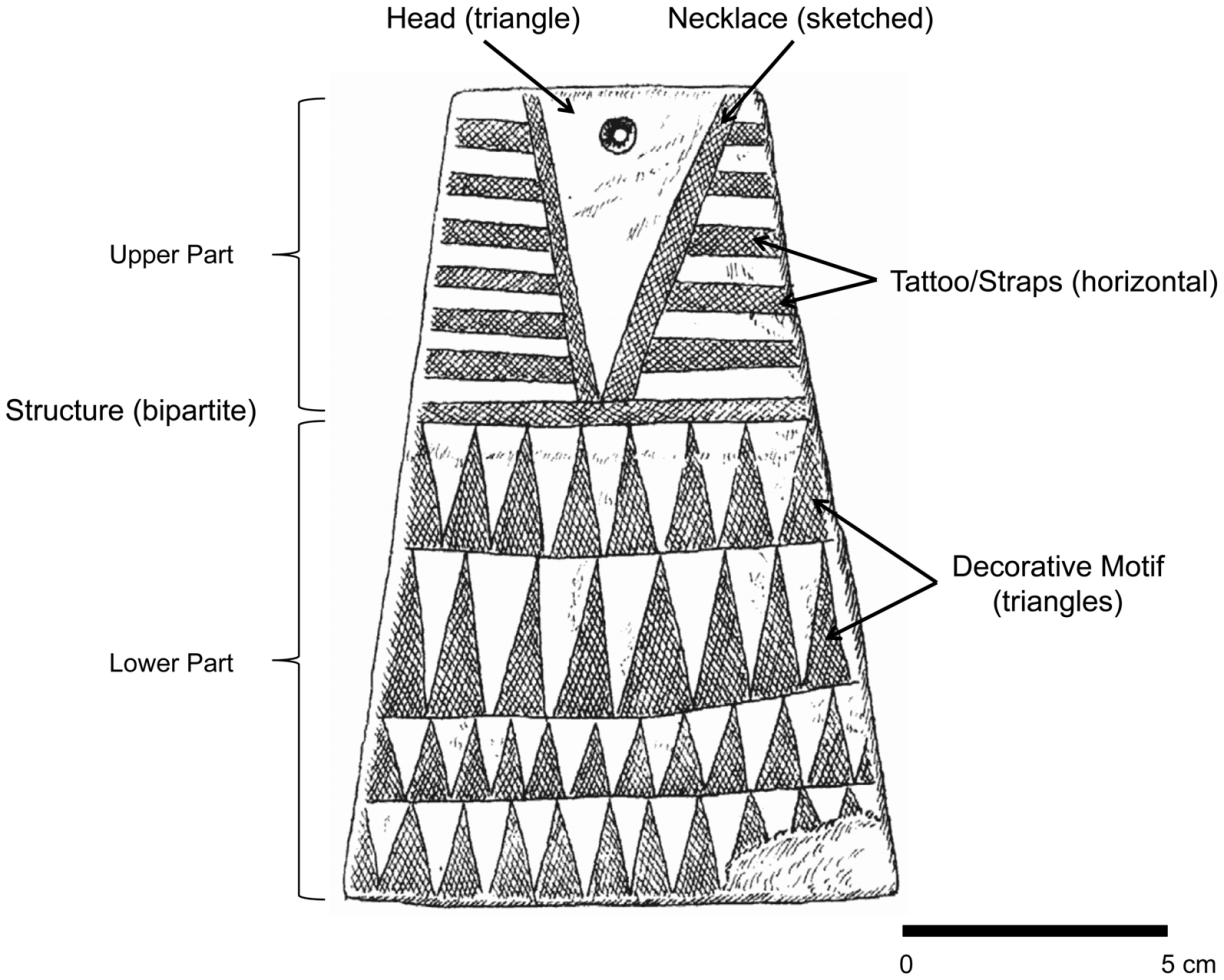
Characters used in the analysis and abbreviations: Decorative Motif (DM), Structure (ST), Traps/Tattoo (TT), Necklace (NK), and Head (H). Terms in parentheses are particular character states for this example (see Fig. 3). After [31].

More recently, Katina Lillios combined two of those functions—ideographic writing and heraldic items—hypothesizing that the majority of the plaques codify genealogical information [2], [24]–[27], whereas others perhaps were relics or specific expressions of several individuals [28], [29]. She proposed that decorative motifs on the lower portion of the plaque—the end opposite the hole (Fig. 2)—identify individual descent groups and that the number of decorative “registers”—the horizontal rows of triangles shown on the specimen in Fig. 2—indicates the generational distance between the deceased and the founding ancestor of his or her lineage. For example, a plaque containing two rows of triangles would connote “a person two generations removed from a founding ancestor [of the ‘triangle’ lineage]…. The increase in register [row] numbers suggests gradual demic diffusion away from a core ‘ancestral’ area over time” ([2], p. 149). Thus plaques with a higher number of rows should be later that those with fewer rows. And, just as with the concentric circles that radiate out from a pebble thrown in a pond, the number of rows should increase with distance from the original center of plaque development, as groups moved outward, carrying the plaque-making tradition with them.

Lillios created a sequence of types based on expected chronological changes in various features of the plaques, with emphasis on decorative motifs on the lower portion of the plaque. She anchored the sequence with plaques containing vertical bands, herringbone designs, or checkerboard patterning (Fig. 1), followed by plaques with zigzag decoration, followed by plaques with chevrons and triangles. Her reasoning was that many of the examples with vertical bands, herringbone designs, and checkerboard patterning appeared to come from the Évora district of southern Portugal, which many Iberian archaeologists consider to be the original heartland of Late Neolithic peoples responsible for the megalithic tombs in which many of the plaques have been found [10], [30], [31]. So her reasoning went, as those peoples moved out from Évora, the plaques they made became increasingly younger in age, ending with triangle designs.

Lillios ([2], pp. 157–158) states that she used both the “proxy method of ordering the plaques” as well as phyletic seriation [32] to “propose a tentative chronological sequencing” of plaques, but she presents no data that would allow us to examine the strength of the sequence. A few comments are in order. First, her “proxy method” is based on using row number as a chronological proxy (fewer rows early, more rows later) and then placing the plaques in sequence based on the number of rows they contain. This method, however, assumes that row number is actually a measure of elapsed time, which is what Lillios was trying to establish in the first place. Thus, any results are tautological. We return to this point later. Second, Lillios did not test the sequence either stratigraphically or against radiometric dates. Admittedly, there are only a small number of published radiocarbon dates available (Table 1), but as we discuss later, they, together with published stratigraphic information, are clear indicators that Lillios's sequence is suspect.

Despite these problems, Lillios's hypothesis has gained considerable weight among archaeologists working on the Iberian Peninsula ([33], [34]; but see [21]). To test her hypothesis, we turned to an evolutionary model—cladogenetic, or branching, evolution—that reflects the nature of evolutionary change, whether in organisms or material culture [35]–[37]. Instead of collapsing all change into a single line of ancestry, as in the model underlying phyletic seriation, the cladogenetic model recognizes that ancestry is bushy, or tree-like. As we detail below, we designed a series of phylogenetic exercises—similar to protocols used on other archaeological materials [36], [38], [39]—to *maximize* the expectations of Lillios's hypothesis. This meant that we weighted *every* experimental protocol and analytical decision in favor of her hypothesis, our rationale being that if we tried every way possible to meet the expectations but could not, then the hypothesis should be rethought.

In summary, our analysis did *not* support Lillios's hypothesis that the plaques are genealogical mnemonic recording systems. We should say that her hypothesis is not supported *in its current form*, meaning that it is her proposed sequence of plaque designs that is unsupported. Our analysis does not negate the possibility that the plaques served as mnemonic devices or some other function tied to “external symbolic storage” [40], [41]. Whatever their purpose, it appears there was a common ideological background to the use of plaques that overlay the southwestern Iberian Peninsula—a cultural system in which plaque design was based on a fundamental core tradition, similar to Swadesh's [42] “morphological kernel” of a language [43], [44], with a number of mutable and variable elements surrounding it.

## Materials

Our data were derived from the online Esprit (Engraved Stone Plaque Registry and Inquiry Tool) database (http://research2.its.uiowa.edu/iberian/index.php) created by Lillios and collaborators [45]. It contains information on over 1400 plaques. Although some archaeologists [46] have claimed that the data set does not contain the total number of excavated plaques, which is likely, we have no reason to think that it is not representative of the variation in decoration that existed across the southwestern Iberian Peninsula.

**Figure 3:**
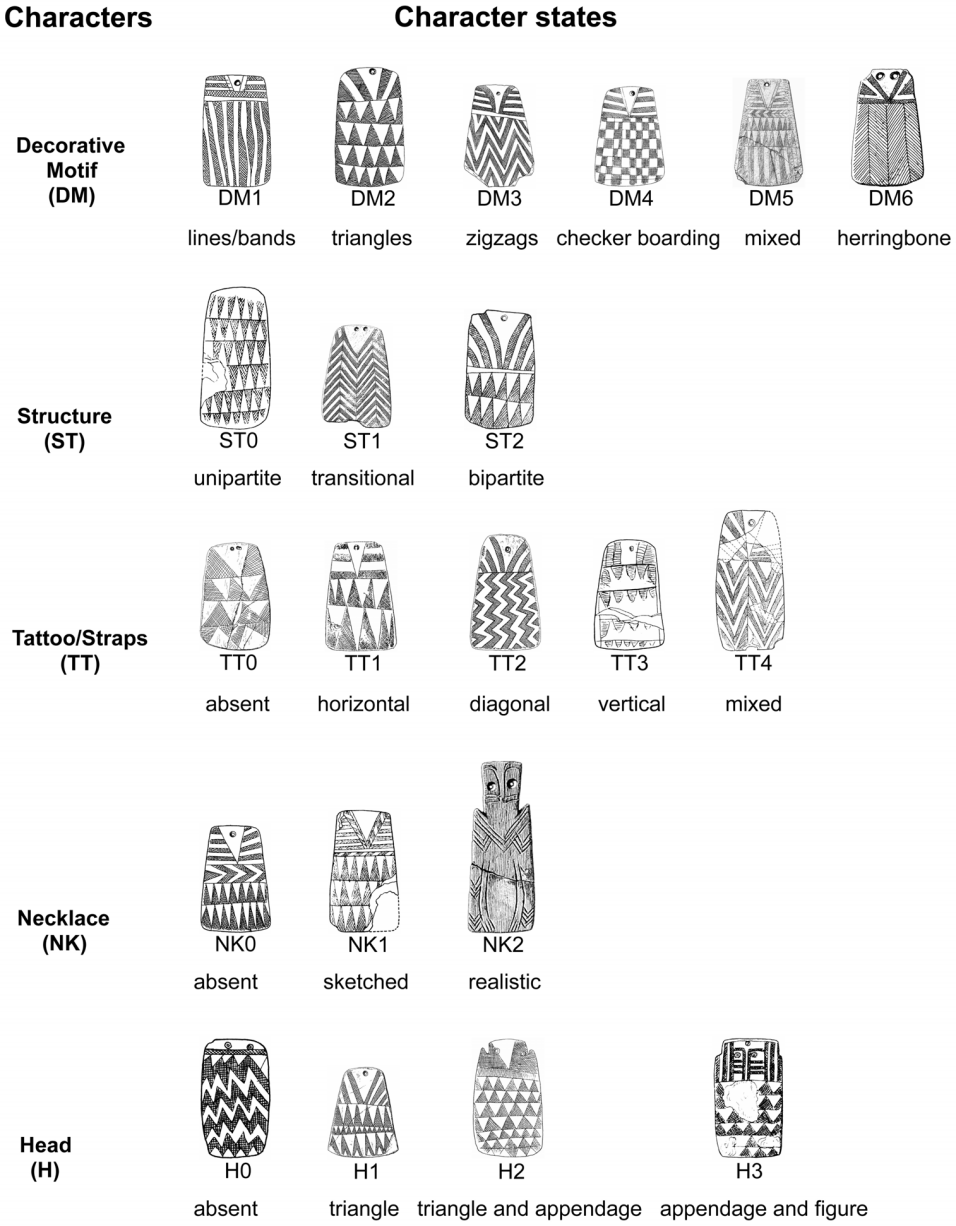
Character states used in the analysis.

Because of inconsistencies in how plaque types have been created by the authors of and contributors to Esprit—not an unusual occurrence in archaeologywe used paradigmatic classification to define analytical classes [35], [47]. In paradigmatic classification, the investigator specifies a priori the characters and character states that are of analytical interest, and each specimen is then classified by linking the state of each character. Any character state can combine with any of the states of the other characters to create a class (taxon). Key to Lillios's hypothesis is the character “base decorative motif” (DM), but five other characters are also proposed to have chronological significance: “structure (ST),” “tattoo straps (TT),” “necklace (NK),” “head motif (H),” and “number of registers (rows).” We excluded the last character because, if we use Lillios's reasoning, it is not an independent variable. As we mentioned above, to her, rows of engraved lines tell us about the *use life* of a plaque, not its *chronological age*. To use our earlier example, a plaque with two rows signifies two generations, whereas a plaque with five rows signifies five generations. The first plaque could have been made many centuries before the second one but did not record as many generations before it was placed in the ground. Fig. 3 shows the possible discrete states of each of the five characters used.

By excluding specimens that were broken, showed evidence of re-engraving, or were not illustrated in the online database, we ended up with a population of 735 plaques. We judged the population to be too large to analyze because of the number of unique classes involved (see below), so we reduced it to 349 specimens using a 5% error and a 99% confidence interval (http://www.med.unne.edu.ar/biblioteca/calculos/calculadora.htm). Those 349 specimens were spread across 81 classes. Table 2 lists the number of specimens by class and Esprit database identification number [45]. For the latter we used the first specimen listed in the database as the class representative. For example, class 1 contains 15 specimens that are identical in terms of character states. We list only one specimen identification number for that class instead of all 15. Table 3 reorders the data in Table 2 to show the number of duplicate cases across the sample. For example, the first row of the table shows that 36 classes contain only one specimen (there are zero duplicate specimens in those 36 classes). The second-to-last row shows that there is one class that contains 27 specimens (one plus 26 duplicates). We can see from Table 2 that that 27-specimen class is class 3.

**Table 2:** Distribution of specimens across the 81 classes.

Class	Esprit Identification	Number of Specimens
1	1	15
2	2	1
3	3	27
4	8	5
5	12	2
6	13	6
7	14	23
8	15	3
11	18	4
12	19	1
13	23	17
14	29	22
15	30	5
16	31	3
18	33	2
20	35	1
21	36	10
24	44	6
25	45	1
26	46	8
32	53	24
39	61	2
41	63	5
42	64	6
44	66	7
45	68	9
50	75	5
56	85	3
59	89	4
91	157	10
94	160	18
96	163	4
97	164	3
107	175	5
111	180	1
114	183	3
138	226	4
146	236	4
150	241	11
151	243	3
155	253	2
161	259	2
169	268	1
174	275	5
177	279	1
180	282	1
189	296	1
211	321	1
219	332	2
222	335	1
225	340	2
244	365	1
249	370	3
271	405	1
304	466	2
313	479	2
321	489	1
328	499	1
330	501	1
342	513	3
344	515	1
355	536	1
361	544	1
401	612	1
415	637	1
420	650	2
432	676	1
438	700	1
442	709	1
478	836	1
483	844	1
497	861	1
498	862	1
616	1074	1
618	1076	1
623	1084	1
638	1109	1
660	1165	1
670	1178	1
681	1191	1
690	1221	1

**Table 3:** Frequency and percentage of classes that have multiple specimens.

Number of Multiple Specimens	Frequency	Percentage of 349 Specimens	Cumulative Percentage of 349 Specimens
0	36	10.3	10.3
1	10	5.7	16.0
2	8	6.9	22.9
3	5	5.7	28.6
4	6	8.6	37.2
5	3	5.1	42.3
6	1	2.0	44.3
7	1	2.3	46.6
8	1	2.6	49.2
9	2	5.7	54.9
10	1	3.1	58.0
14	1	4.3	62.3
16	1	4.9	67.2
17	1	5.1	72.3
21	1	6.3	78.6
22	1	6.6	85.2
23	1	6.9	92.1
26	1	7.7	99.8
Total	81	99.8	

**Figure 4:**
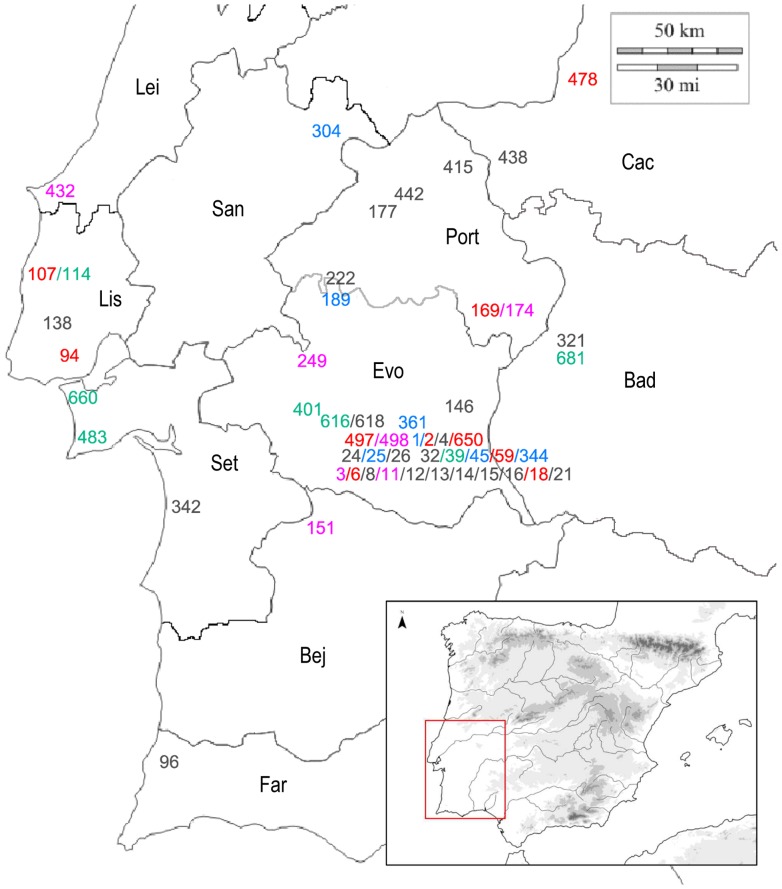
Distribution map of the four data sets. Sample 1: red; sample 2: green; sample 3: blue; and sample 4: pink. Gray numbers reference plaques that belong to more than one sample. The names of Portuguese districts are Leiria (Lei), Lisboa (Lis), Setúbal (Set), Beja (Bej), Faro (Far), Évora (Evo), and Portalegre (Port), and the Spanish provinces are Cáceres (Cac) and Badajoz (Bad).

Because the tree-building computer program we used—PAUP* 4.0 [48] (see below)—could not accommodate that number of classes, we took a weighted random sample (with replacement [SPSS v. 20]) from the 81-class sample to create 4 samples of 20 classes each. (The weight of each class was determined by the number of specimens in it.) Table 4 lists the classes in each sample and their character states; Fig. 4 shows the distribution of classes geographically.

**Table 4:** Data matrix for the four samples.

Class	ID^1^	DM^2^	ST^3^	TT^4^	NK^5^	H^6^
Sample 1						
2 Evo^7^	2	1	2	2	1	1
6 Evo	13	3	2	0	1	1
12 Evo	19	2	2	0	1	2
13 Evo	23	2	2	1	1	1
14 Evo	29	3	2	1	1	1
15 Evo	30	5	2	2	0	1
18 Evo	33	3	0	0	0	2
26 Evo	46	5	0	0	0	0
32 Evo	53	2	2	1	0	1
59 Evo	89	4	2	1	0	1
94 Lis	160	2	2	0	0	1
96 Far	163	2	2	0	1	1
107 Lis	175	5	2	0	0	1
169 Port	268	5	2	0	1	2
222 Port	335	2	1	3	0	1
342 Set	513	1	2	1	1	1
415 Port	637	6	0	0	1	1
420 Evo	650	2	0	0	0	2
478 Cac	836	1	0	0	0	3
497 Evo	861	3	1	4	0	1
Sample 2						
4 Evo^7^	8	3	2	4	0	1
8 Evo	15	1	2	2	0	1
12 Evo	19	2	2	0	1	2
21 Evo	36	4	2	2	0	1
26 Evo	46	5	0	0	0	0
32 Evo	53	2	2	1	0	1
39 Evo	61	2	2	3	0	1
114 Lis	183	3	1	2	0	1
138 Lis	226	3	2	0	0	0
177 Port	279	2	1	0	0	0
222 Port	335	2	1	3	0	1
321 Bad	489	3	2	2	0	0
401 Evo	612	5	1	1	1	1
415 Port	637	6	0	0	1	1
442 Port	709	2	1	0	1	2
483 Set	844	3	2	1	0	0
616 Evo	1074	3	2	0	0	1
618 Evo	1076	6	2	1	0	1
660 Set	1165	3	1	1	1	1
681 Bad	1191	2	2	2	1	1
Sample 3						
1 Evo^7^	1	3	2	1	0	1
12 Evo	19	2	2	0	1	2
14 Evo	29	3	2	1	1	1
15 Evo	30	5	2	2	0	1
16 Evo	31	3	0	0	1	1
21 Evo	36	4	2	2	0	1
24 Evo	44	2	2	0	0	2
25 Evo	45	6	1	0	0	1
32 Evo	53	2	2	1	0	1
45 Evo	68	5	2	1	0	1
138 Lis	226	3	2	0	0	0
146 Evo	236	6	2	1	1	1
177 Port	279	2	1	0	0	0
189 Evo	296	2	1	0	1	1
304 San	466	5	2	0	0	2
321 Bad	489	3	2	2	0	0
344 Evo	515	1	2	3	0	1
361 Evo	544	3	2	0	2	2
438 Cac	700	2	2	1	0	0
442 Port	709	2	1	0	1	2
Sample 4						
3 Evo^7^	3	2	2	2	0	1
4 Evo	8	3	2	4	0	1
11 Evo	18	2	0	0	0	1
13 Evo	23	2	2	1	1	1
14 Evo	29	3	2	1	1	1
16 Evo	31	3	0	0	1	1
24 Evo	44	2	2	0	0	2
32 Evo	53	2	2	1	0	1
96 Far	163	2	2	0	1	1
146 Evo	236	6	2	1	1	1
151 Bej	243	3	0	0	0	0
174 Port	275	5	2	0	0	0
177 Port	279	2	1	0	0	0
249 Evo	370	1	0	0	0	0
342 Set	513	1	2	1	1	1
415 Port	637	6	0	0	1	1
432 Lei	676	3	1	2	1	1
438 Cac	700	2	2	1	0	0
498 Evo	862	1	2	0	1	1
618 Evo	1076	6	2	1	0	1

1 ID = specimen number in the ESPRIT database [45].

2 DM = decorative motif.

3 ST = structure.

4 TT = tattoo straps.

5 NK = neck.

6 H = head.

**Figure 5:**
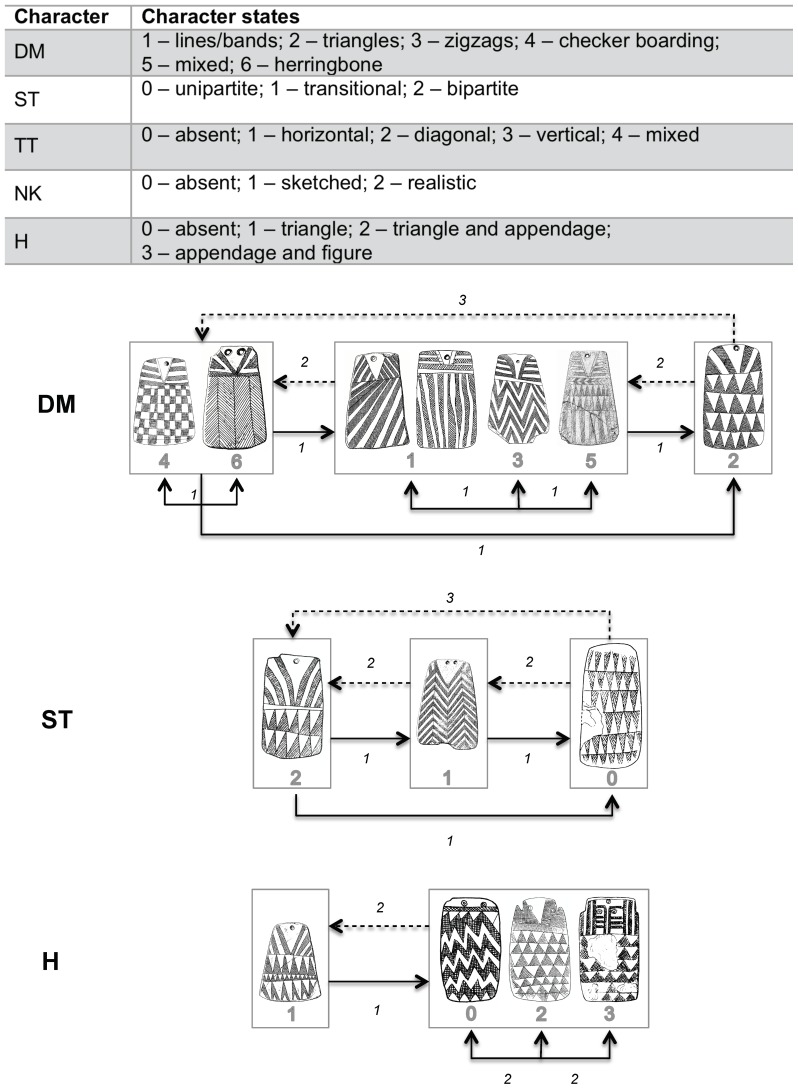
Characters and character states used in the analysis reported here, together with polarities and cost of transitions between states for characters DM, ST, and H, with polarity set by Lillios's hypothesis. Plaques appearing within one rectangle have the same polarity. Continuous lines indicate less-costly transitions, and dashed lines indicate more-costly transitions. Numbers indicate cost values for corresponding transitions in exercises 3 and 4 (Table 5).

7 Abbreviations to the right of the class numbers refer to the geographic provinces shown in Fig. 5.

## Method

Phylogenetic reconstruction is the main method used in biology to construct testable hypotheses of ancestor–descendant relationships [49]–[52]. It has also begun to see wide usage in archaeology [37]–[39], [53]–[58] and other studies of material culture [59]–[67]. As Riede ([58] p. 799) points, cultural phylogenetics

has advantages over traditional typological approaches in that a given phylogeny constitutes a quantitative hypothesis of the historical relatedness among the chosen units of analysis…. Such

hypotheses can then be evaluated statistically and in relation to external datasets, such as stratigraphic, geographical or radiocarbon dating information. While a phylogenetic quantification of material culture relations alone can reveal important new insights in its own right, phylogenies can also be used in additional comparative analyses.

Phylogenetics is based on a model of descent with modification in which new taxa arise from the bifurcation of existing ones. Phylogenetic relationships are defined in terms of relative recency of common ancestry: Two taxa are deemed to be more closely related to one another than either is to a third taxon if they share a common ancestor that is not also shared by the third taxon. The evidence for exclusive common ancestry is the sharing of evolutionarily novel, or derived, character states, termed *synapomorphies*.

Various methods have been used for phylogenetic inference, each based on different models and each having its own strengths and weaknesses [68]–[72]. The one we used, maximum parsimony, is based on a model that seeks to identify the least number of evolutionary steps required to arrange the taxonomic units under study. In simplest form, the method consists of four steps:

1. Generation of a data matrix that shows the states of the characters exhibited by each taxon.
2. Establishment of direction (polarity) of evolutionary change among the states of each character. One method for doing this is outgroup analysis [73], which entails examining a close relative of the study group. When a character occurs in two states among the study group, but only one of the states is found in the outgroup, the principle of parsimony is invoked (see above), and the state found only in the study group is deemed to be evolutionarily novel with respect to the outgroup state.
3. Construction of a branching diagram of relationships for each character by joining the two most derived taxa—those at the branch tips of a tree—and then successively connecting each of the other taxa according to how derived they are. Ideally, the distribution of character states among the taxa will be such that all the character trees imply relationships among the taxa that are congruent with one another. Normally, however, a number of the character trees will suggest relationships that are incompatible—a phenomenon known as *homoplasy*. This problem is overcome through the fourth step:
4. Construction of an ensemble tree that is consistent with the largest number of characters and therefore requires the smallest number of homoplasies to account for the distribution of character states among the taxa. We refer to such a tree as the “most parsimonious” solution. Parsimony trees are evaluated on the basis of the minimum number of character-state changes required to create them, without assuming a priori a specific distribution of trait changes. This compensates for the process pathways, biases, and random variation that characterize “cultural transmission” [74]–[76]. It is worth underscoring that trees are *hypothetical* statements of relatedness, “given the model and parameters used” ([68] p. 189), not irrefutable statements of precise phylogenetic relationships.

Numerous techniques are available for measuring the goodness of fit between a data set and a given tree, with the consistency index (CI), the retention index (RI), and the rescaled consistency index (RC) being the most commonly used. The CI measures the relative amount of homoplasy in a data set but is dependent on the number of taxa. Thus, the expected CI for a given tree must be assessed relative to the number of taxa used in the analysis [77]. The RI measures the number of similarities in a data set that are retained as homologies in relation to a given tree. It is insensitive to both the presence of derived character states that are present in only a single taxon and the number of characters or taxa employed. Thus, it can be compared among studies. The rescaled consistency index (RC) is the product of the consistency index and the retention index. Indices range from zero, which indicates a lack of fit between a tree and the data set used to generate it, to 1.0, which represents a perfect fit.

Our phylogenetic analysis consisted of four exercises (Table 5), each of which was carried out on each of the four samples listed in Table 4. Each exercise searched for the best-supported tree using the same tree-building methods and character/character-state parameters (Table 4). We used the “parsimony heuristic search” in PAUP*. All searches were carried out using the stepwise-addition strategy for the addition of classes, with a simple addition sequence and keeping only one tree at every step; the tree bisection and reconnection method, with the branch-swapping algorithm in relation to the tree rearrangements; and a maximum set of 100 for the initial trees. The following scores were extracted from all searches: number of trees, length of trees, consistency index (CI), retention index (RI), and rescaled consistency index (RC). We generated three kinds of consensus trees—strict, semi-strict, and majority-rule—to reconcile different outcomes. We also generated bootstrap trees using the following parameters: 100 bootstrap replicates; simple weighting; randomly starting seed; parsimony optimality criterion; and 500 saved trees in each bootstrap replicate step.

**Table 5:** Conditions of the four phylogenetic exercises.

Exercise	Sample	Methods	Parameters	Outgroup
1	1	PHS^1^/ACT^2^/BT^3^	UC^4^/US^5^	UNRT^6^
1	2	PHS/ACT/BT	UC/US	UNRT
1	3	PHS/ACT/BT	UC/US	UNRT
1	4	PHS/ACT/BT	UC/US	UNRT
2	1	PHS/ACT/BT	WC^7^/US	UNRT
2	2	PHS/ACT/BT	WC/US	UNRT
2	3	PHS/ACT/BT	WC/US	UNRT
2	4	PHS/ACT/BT	WC/US	UNRT
3	1	PHS/ACT/BT	UC/OS^8^	ROOT^9^ (no. 59)
3	2	PHS/ACT/BT	UC/OS	ROOT (no. 21)
3	3	PHS/ACT/BT	UC/OS	ROOT (no. 146)
3	4	PHS/ACT/BT	UC/OS	ROOT (no. 618)
4	1	PHS/ACT/BT	WC/OS	ROOT (no. 59)
4	2	PHS/ACT/BT	WC/OS	ROOT (no. 21)
4	3	PHS/ACT/BT	WC/OS	ROOT (no. 146)
4	4	PHS/ACT/BT	WC/OS	ROOT (no. 618)

1 Parsimony Heuristic Search.

2 All Consensus Trees (strict, semistrict, and 50% majority rule).

3 BooTstrap.

4 Unweighted Characters.

5 Unordered States.

6 UNRooTed trees (no predefined outgroup).

7 Weighted Characters.

8 Ordered States.

9 ROOTed trees (predefined outgroup).

Certain characters can be hypothesized as being more important than others in determining phylogenetic relationships, and thus more analytical *weight* can be placed on them. As mentioned, our goal was to stack the deck in favor of Lillios's hypothesis, in which character DM plays a crucial role, so for exercises 2 and 4 (Table 5), we assigned it a weight of 2, whereas characters ST, TT, NK and H were each assigned a weight of 1.

Character states can also be *ordered*, which means there are defined pathways that a character transformation can take [78]. Thus, for example, it may be the case that evolutionary “laws” dictate that an organism can lose or gain only one toe at a time. It could move from five toes to four toes, or vice versa, but never from five to three or from two to four. The character “number of toes,” then, is said to have ordered character states. In reality, an ordered transformation series is a *hypothesis* about a particular pathway because rarely will we know absolutely what is possible in nature. Lillios's hypothesis assumes that the lower portion of the plaques, containing character DM, represents lineage affiliations, whereas the number of rows represents the generations back to the founding ancestor of the lineage [2], [24], [25]. With respect to character DM, Lillios suggests that plaques with checkerboard (DM_4_) and herringbone (DM_6_) designs are ancestral to plaques with other decorative designs, given that the former are more limited to her suspected core area of plaque manufacture and use (the Évora district of southern Portugal).

For exercises 3 and 4 (Table 5), we assigned costs to changes that violated the order of character states indicated by Lillios's hypothesis. As shown in Fig. 5, transformations from supposed ancestral character states to derived states—say, from herringbone (DM_6_) to zigzag (DM_3_)—have the lowest cost (1), meaning that they have evolved in the manner Lillios suggested. At the other end of the spectrum, a transformation from a derived state to the *original* ancestral state—say, from triangle (DM_2_) to herringbone (DM_6_) or from unipartite (ST_0_) to bipartite (ST_2_)—has a cost of 3. Any transformation between a derived state and one immediately preceding it (an intermediate state)—say, from triangles (DM_2_) to zigzag (DM_3_)—has a cost of 2, as does any transformation between derived states of character H. Here, Lillios proposed that the inverted triangular head (H_1_) was immediately ancestral to all three other states (H_0_, H_2_, and H_3_), meaning she identified no intermediate states between the ancestral state and the three derived states. We find it difficult to believe, however, that there are no intermediate states and view the situation as a polytomy—an unresolved (nondichotomous) branching episode. If so, there are more possible reversals than there are nonreversals and thus we gave all changes among H_0_, H_2_, and H_3_ a cost of 2 [79]. If anything, this move stacked the deck even further in favor of Lillios's hypothesis, given that the best two trees (see below) both contained ordered character states. We did not order characters TT and NK because Lillios's hypothesis is unclear as to their chronological ordering.

Two exercises were carried out with outgroups (ROOT in Table 5) and two without outgroups (UNRT in Table 5). We used classes 21, 59, 146, and 618 as outgroups because they display all or most of the presumed ancestral states in Lillios's hypothesis—again, a deliberate decision to maximize polarity in favor of the hypothesis. In summary, exercise 1 used unweighted characters, unordered character states, and unrooted trees; exercise 2 used weighted characters, unordered character states, and unrooted trees; exercise 3 used unweighted characters, ordered character states, and rooted trees; and exercise 4 used weighted characters, ordered character states, and rooted trees (Table 5).

## Results

Table 6 presents the following scores for each heuristic search: number of most-parsimonious trees returned, branch length of trees, consistency index (CI), retention index (RI), and rescaled consistency index (RC). The number of most-parsimonious trees obtained in the exercises is high, running into the tens of thousands. The CI, RI, and RC show strong differences among the exercises. In particular, the CI decreases dramatically between exercises 1 and 2, with a mean of 0.56, and exercises 3 and 4, with a mean of 0.16. The RI, however, decreases from 0.65 to only 0.52. The RC indicates the same trend as the CI: Exercises 1 and 2 have a mean of 0.36, whereas exercises 3 and 4 have a mean of 0.08.

**Table 6:** Parsimony heuristic search scores.

Exercise	Sample	Number of Trees	Length of Trees	Consistency Index	Retention Index	Rescaled Consistency Index
1	1	69800	25	0.600	0.655	0.393
1	2	54368	26	0.538	0.636	0.343
**1** 1	**3**	**21545**	**24**	**0.583**	**0.667**	**0.389**
1	4	73400	24	0.500	0.625	0.312
2	1	73400	33	0.606	0.649	0.393
2	2	71100	33	0.576	0.659	0.379
**2**	**3**	**71600**	**32**	**0.594**	**0.658**	**0.391**
2	4	69100	31	0.516	0.625	0.323
3	1	72900	29	0.172	0.467	0.080
**3**	**2**	**72100**	**27**	**0.185**	**0.532**	**0.099**
3	3	71600	27	0.185	0.500	0.093
3	4	71600	26	0.154	0.511	0.079
4	1	70900	36	0.139	0.516	0.072
**4**	**2**	**72100**	**34**	**0.147**	**0.554**	**0.081**
4	3	72100	34	0.147	0.532	0.078
4	4	72900	31	0.129	0.565	0.073

1 Bold indicates samples with the best general scores for every exercise.

The RI scores from all the exercises indicate that the data set has some consistency and phylogenetic structure. The sharp contrast observed for CI and RC values between the first two and the last two exercises indicates a significant difference related to the methodological parameters applied, specifically the switch from parameter US (unordered states) to parameter OS (ordered-states). In contrast, parameter WC, which implements different weights for a couple of characters, has no influence on the values. This indicates that the suspected order of character states in the hypothesis is inaccurate.

**Figure 6:**
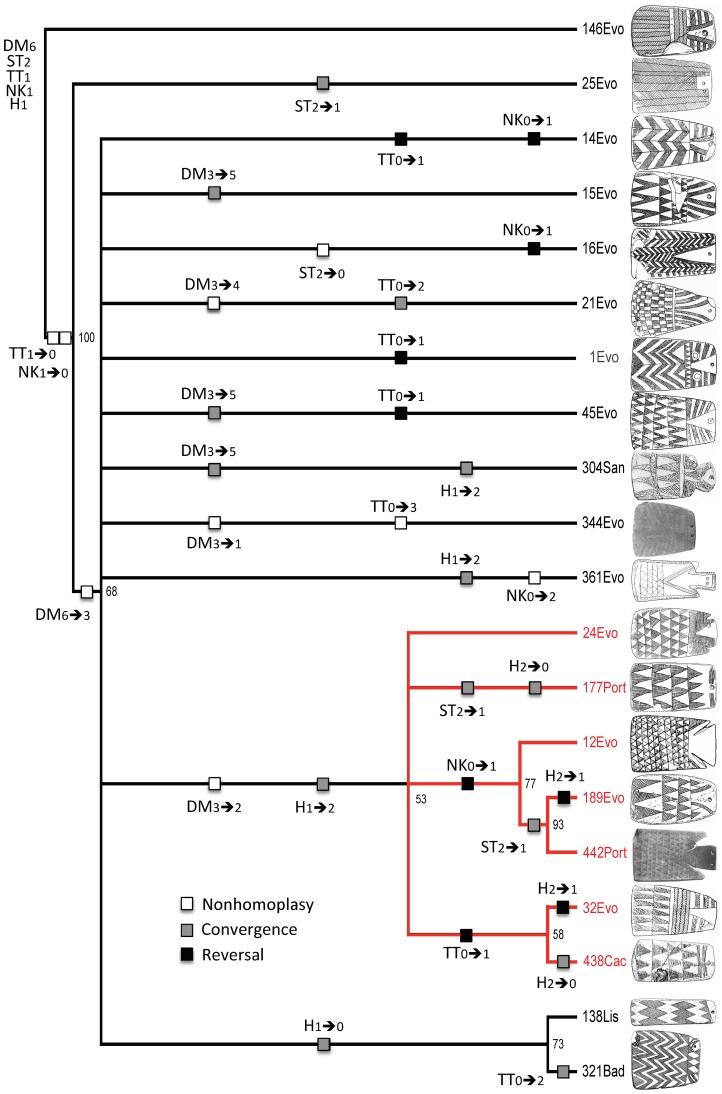
Fifty-percent majority-rule consensus tree from exercise 2, sample 3. The tree, which uses weighted characters but unordered character states, has a CI of 0.594, an RI of 0.658, and an RC of 0.391. When generated by PAUP, the tree was unrooted, but it subsequently was rooted with class 146 to resolve the topology in favor of Lillios's hypothesis. Numbers at nodes are bootstrap values.

**Figure 7:**
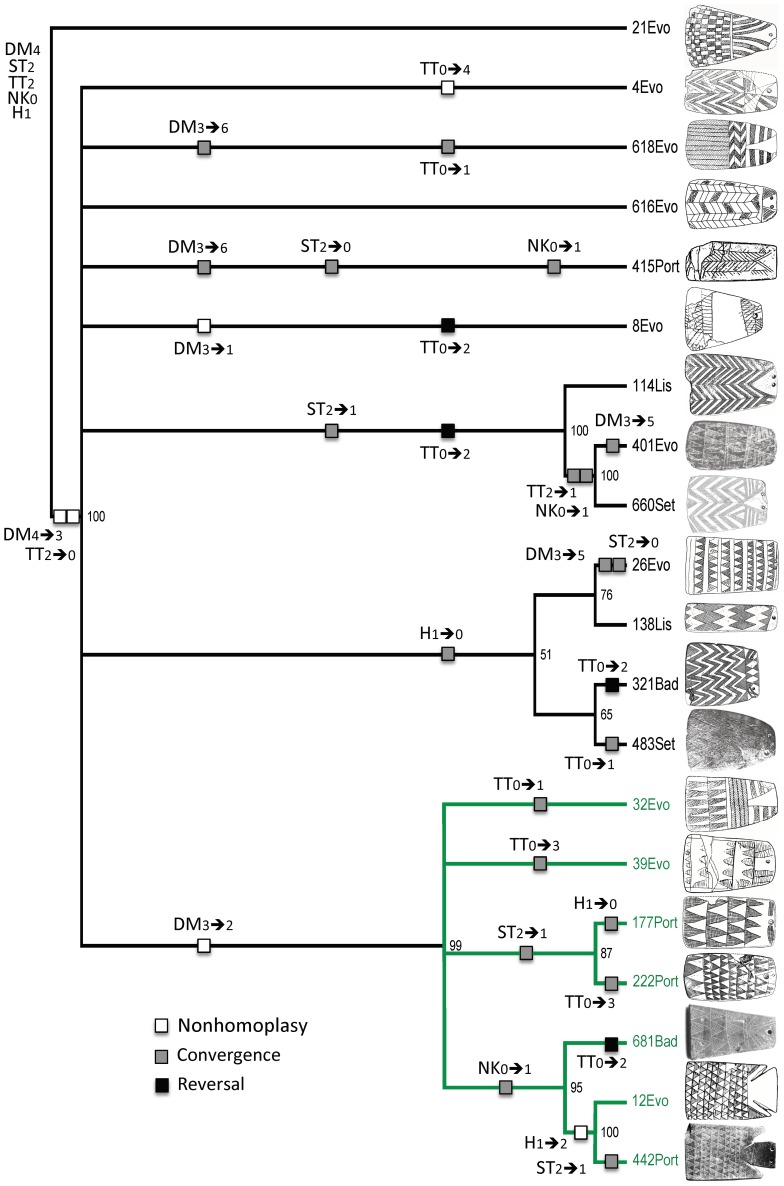
Fifty-percent majority-rule consensus tree from exercise 4, sample 2. The tree, which uses weighted characters and ordered character states, has a CI of 0.147, an RI of 0.554, and an RC of 0.081. Numbers at nodes are bootstrap values.

We next created four trees per exercise and sample—three consensus trees (strict, semistrict, and 50% majority rule) and a bootstrap tree (Table 5). The result was 64 trees (16×4). We then reduced the number of trees to two in order to focus on those that *best fit* the expectations of Lillios's hypothesis (Table 6). Those two trees come from exercise 2 (sample 3)—termed the “2/3 tree” (Fig. 6)—and exercise 4 (sample 2)—the “4/2 tree” (Fig. 7). Both are 50% majority-rule trees [80]; the 2/3 tree was unrooted, and the 4/2 tree was rooted. When PAUP* creates rooted trees, it sets polarity—the direction of character-state change—using outgroups selected by the analyst. The 4/2 tree (Fig. 7) was rooted using class 21 (Table 5), the class in the sample that displayed the highest number of presumed ancestral states in Lillios's hypothesis. When PAUP* creates unrooted trees, its default is to start with the first taxon in the input list and build from there. PAUP* constructed the 2/3 tree using class 1 as a starting point. After examining the tree, however, we went a step further in favoring the hypothesis. We swapped class 146 for class 1 because it is another class that displays all or most of the presumed ancestral states in Lillios's hypothesis. The CI, RI, and CR remained unaffected.

In general, the two trees have large sections that exhibit poor structural and topological resolution. Both have some large polytomies (unresolved branches) close to their roots. In the 2/3 tree (Fig. 6), the basal branching episode creates class 25, but it also creates nine unresolved branches (class 14 through class 361). The 4/2 tree (Fig. 7) has a basal polytomy, five branches of which (class 4 through class 8) are completely unresolved. Both trees also exhibit sections where relationships are more resolved. The 2/3 tree (Fig. 6), for example, contains a seven-class clade, shown in red, with considerable branching structure. The 4/2 tree (Fig. 7) also contains a seven-class clade, shown in green, that contains two smaller, multiclass clades.

Given the overall lack of deep structure, it is not surprising that no character is free of homoplasy. In the 2/3 tree (Fig. 6), only 9 of the 32 character-state changes are nonhomoplastic, and only 4 of those are synapomorphic (TT_1→0_ and NK_1→0_ at the base of the tree; DM_6→3_ at the next node up; and DM_3→2_ in the seven-class red clade). The character with a balance between nonhomoplastic (not necessarily synapomorphic) and homoplastic change is DM, which has four instances of the former and three of the latter. There are several reversals to ancestral states as a result of convergence. In the 4/2 tree (Fig. 7), only six changes are nonhomoplastic. Of these, four are synapomorphies (DM_4→3_ and TT_2→0_ at the base; DM_3→2_ in the seven-class green clade; and H_1→2_ in the clade comprising classes 12 and 442). All characters exhibit at least one instance of homoplasy.

## Discussion

Synapomorphies help resolve trees, but here our interest is primarily in the polarity of specific character states: How well does polarity meet the expectations of Lillios's hypothesis? We identified the following expectations:

1. With respect to DM, states other than DM_4_ and DM_6_—the presumed ancestral states (Fig. 5)—should be located nearer to the branch tips;
2. ST_2_ (bipartite structure) is ancestral to ST_1_ (transitional) and ST_0_ (unipartite); and
3. H_1_ (inverted triangles) is presumed to be ancestral to H_0_ (no head), H_2_ (triangles and appendages), and H_3_ (appendages and figurative features).

**Figure 8:**
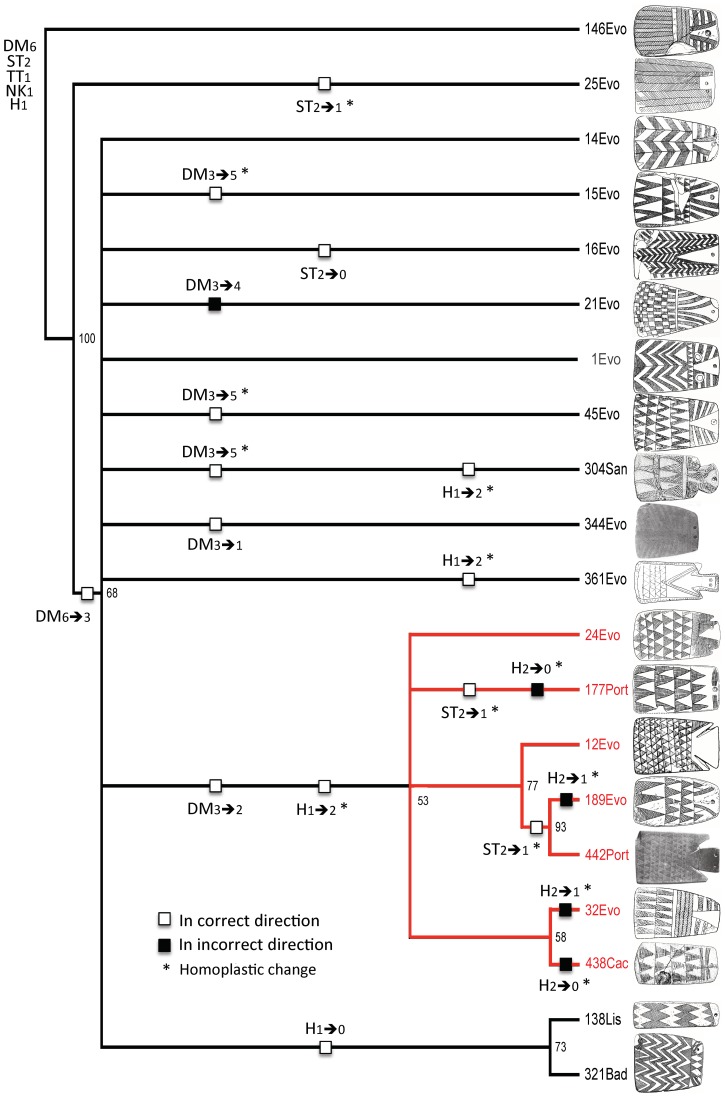
Fifty-percent majority-rule consensus tree from exercise 2, sample 3 (the 2/3 tree) showing character-state changes states according to the assumed polarities for characters DM, ST, and H. Changes of states for characters TT and NK do not appear here because Lillios's hypothesis makes no assumptions about polarity. Numbers at nodes are bootstrap values.

**Figure 9:**
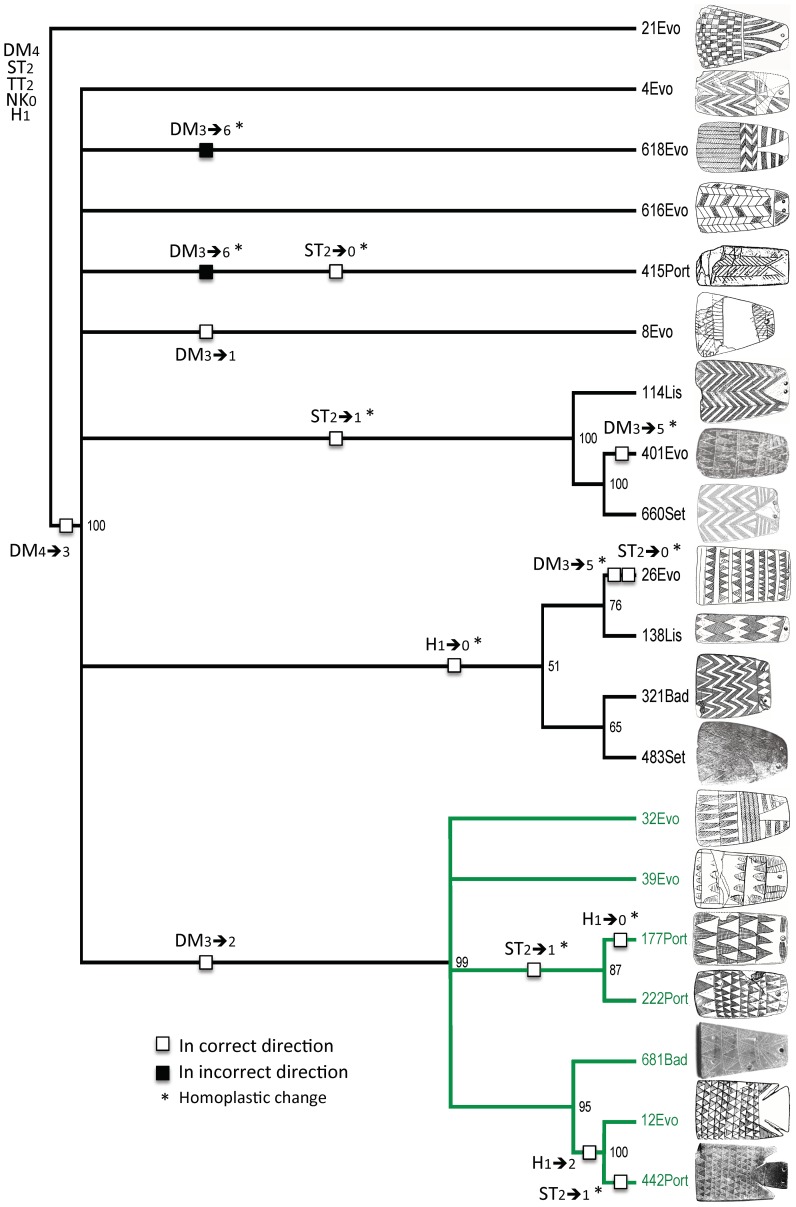
Fifty-percent majority-rule consensus tree from exercise 4, sample 2 (the 4/2 tree) showing character-state changes states according to the assumed polarities for characters DM, ST, and H. Changes of states for characters TT and NK do not appear here because Lillios's hypothesis makes no assumptions about polarity. Numbers at nodes are bootstrap values.

To assess how well the expectations are met, we created the trees shown in Figs. 8 and 9. With two exceptions, the character-state transformations are the same as in Figs. 6 and 7, but they have been converted to binary states in which character-state changes either meet or do not meet expectations. The two exceptions are character states for NK and TT because, although Lillios [2] believes they have chronological significance, her hypothesis is silent as to their polarity. Based on its location on trees 2/3 and 4/2 (Figs. 6 and 7), character NK has no clear nonrandom patterning in the former, and it has the direction NK_0→1_ in the latter (three instances of convergence). With respect to character TT, there is no clear, nonrandom patterning.

How do the expectations fare in the 2/3 tree, which has weighted characters and unordered character states (Fig. 8)? In general, the majority of changes appear in the direction Lillios [2] suggested. Of the 19 changes, only 5 show an unexpected polarity, indicated by black boxes. Nevertheless, 13 are homoplastic changes, which are indicated by asterisks. Specific expectations are considered below:

1. Only one of the seven changes in character DM has an unexpected direction (three are homoplastic changes), the transition DM_3→4_ (zigzags→checkerboard) in class 21. The majority of the changes (five out of seven) are located on terminal branches and thus contribute nothing to the tree structure. Only two synapomorphies are consistent with the expected polarity: DM_6→3_ (herringbone→zigzags) in the large polytomy and then DM_3→2_ (zigzags→triangles), which creates the clade of seven classes.
2. Character ST has four changes that fit the expected polarity (three are homoplastic), and none is a synapomorphy. The ancestral state (ST_2_) is highly conserved, appearing in 15 of the 20 classes.
3. Character H exhibits eight changes (all involve homoplasy), four of them in the hypothesized direction and another four in the opposite direction, with repeated transitions H_2→0_ (triangle & appendage→absence) and H_2→1_ (triangle & appendage→triangle). In addition, the two H_2→1_ reversals are located in unexpected places along the tree. Instead of appearing close to the basal node (H_1_ is the ancestral state), they are at the branch tips of classes 32 and 189. Also, the ancestral trait (H_1_) is highly conserved, appearing in 15 of the 20 classes.

How do the expectations fare in the 4/2 tree, which has weighted characters and ordered character states (Fig. 9)? At first glance, portions of this tree also seem consistent with expected polarity. Only two of the 15 character-state changes are unexpected, although only four are nonhomoplastic. Specific expectations are considered below:

1. Five of the seven changes in character DM exhibit the expected polarity, but two of those are homoplastic. The only two synapomorphies that are consistent with the expected polarity are DM_4→3_ (checkerboard→zigzags) in the basal node and DM_3→2_ (zigzags→triangles), which creates the green clade.
2. All seven changes in character ST have the expected polarity, but all of them are homoplastic. Also, the ancestral state (ST_2_) is very conserved, remaining in 12 of the 20 classes.
3. The three changes in character H meet the expected polarity, but two are homoplastic. The one synapomorphy—H_1→2_ (triangle→triangle & appendage)—sorts only classes 12 and 442. The ancestral state (H_1_) is also highly conserved, remaining in 13 of the 20 classes.

What does the combined topology of two trees tell us about Lillios's hypothesis, especially in combination with radiocarbon dates and stratigraphic information? With respect to character DM, the data might appear at first glance to support Lillios's prediction that plaques with herringbone and checkerboard decoration at the bottom may be the oldest forms. However, there are several reversals (DM_3→4_ [zigzags→checkerboard] and DM_3→6_ [zigzags→herringbone]) in both trees, which undermine the suspected relative late position of plaques containing zigzag decoration. Also, most changes in character DM occur at the branch tips in both trees, which reduces the consistency of this presumptive positive result (recall that DM was weighted in the heuristic searches).

Radiocarbon dates and stratigraphic information also call into serious question the proposed sequence, with several of the oldest dated plaques exhibiting the triangle motif at their base (DM_2_), as, for example, at Cova das Lapas I, in the district of Leiria, with a radiocarbon date of 4550±60 _B.P._ (3238–3108 _B.C._ [1 sigma]) [20], and the oldest level of Anta da Horta, in the Portalegre district of Portugal [81], with a radiocarbon date of 4480±40 _B.P._ (3332–3214 _B.C._ [1 sigma]) [81] (Table 1). According to Lillios's hypothesis, triangles should be the most derived character state. Conversely, the checkerboard motif (DM_4_), supposedly the most ancestral in the suggested sequence of character states, is a late occurrence at Olival da Pega 2b, in the district of Évora, with three calibrated dates [82] that average 2830 _B.C._ (Table 1). With respect to stratigraphic positioning, plaques from the earliest levels at Anta da Horta exhibit the triangle motif (DM_2_) but so do those from later levels. There also are late plaques that exhibit zigzag motif (DM_3_), when, according to the hypothesis, they should be older than plaques with triangles.

With respect to character ST, character state ST_2_ (bipartite) appears to be ancestral to ST_1_ (transitional) and ST_0_ (unipartite)—in line with predictions. In fact, the bipartite structure (ST_2_) is highly conserved. The few relevant dates and available stratigraphic evidence support the conservatism in ST_2_ as well as the derived nature of ST_0_. Whereas dated bipartite (ST_2_) plaques occur (in Cova das Lapas I, and Sala n° 1) at least throughout the period 4550±60 _B.P._ to 4140±110 _B.P._ (3238–3108 _B.C._, and 2876–2618 _B.C._ [1 sigma]), dated unipartite (ST_0_) plaques occur only in the early part of the third millennium (from 4270±40 _B.P._ [2917–2877 _B.C._ 1 sigma] in Anta de STAM-3). At Anta da Horta, all of the plaques in the oldest level are bipartite (ST_2_), and the majority of plaques in the later levels are unipartite (ST_0_).

Recall that predictions relative to character H are not well met in the 2/3 tree (Fig. 8), but they are, broadly, in the 4/2 tree (Fig. 9). There, the inverted-triangle head (H_1_) is the ancestral character state, and appendages (H_2_) and plain (H_0_) are the derived states. H_1_ is highly conserved in the 4/2 tree, and radiocarbon and stratigraphic data bear this out. At Anta da Horta [81], one of the two earliest plaques displays character-state H_1_ and the other H_2_. Plaques from the latest levels exhibit states H_0_ and H_3_. At Olival da Pega 2b [82], H_2_ lasts throughout the sequence, becoming associated with other character states in the later levels.

## Conclusions

The overall implications of Lillios's hypothesis with respect to the evolutionary history of stone plaques on the Iberian Peninsula are not met by a phylogenetic model, even when the two best trees—one with weighted characters (the 2/3 tree) and the other with weighted characters and ordered character states (the 4/2 tree)—are considered. There are at least three possible causes for the poor and arbitrary topology of large sections of both trees, with numerous polytomies and instances of homoplasy:

1. The people who created the plaques were free to use any of the possible states in the design palette at any time and any place. If this were the case, however, we would expect to see unlimited and totally random character-state reversals, which is not the case.
2. There was a high rate of cultural borrowing or horizontal transfer of information among populations scattered across the southwestern Iberian Peninsula, which tended to swamp most of the phylogenetic signal. This would mean that different genealogical and heraldic clans (according to Lillios) shared and transferred much of the information reflected in this material culture, an assumption that would run counter to the hypothesis that the plaques were linked to specific lineages and/or clans.
3. There was a common ideological background (whether religious, apotropaic, and the like) to the use of plaques that overlay the southwestern Iberian Peninsula. This would entail a cultural system in which plaque design was based on a fundamental core idea, with a number of mutable and variable elements surrounding it.

We suspect number 3 was the case, at least in part. It seems reasonable to conclude that most cultures have a conservative “core tradition”—similar to Swadesh's [42] “morphological kernel” of a language [43], [44]. The question is whether we can identify it [59]. We might start by examining how archaeologists have long viewed traditions, going back to Willey's [83] definition: a line or related lines of development through time within the confines of a certain technique or constant. A tradition includes broad categories of such things as plaque designs that undoubtedly have value in expressing historical relationships when the relationships are confined to the geographic boundaries of cultures. We thus should not be surprised that some of the phylogenetic trees derived from the plaque data exhibit internal branching because cultural evolution is, after all, a process that produces cladogenesis [56].

Nor should we be surprised that within the broad trees comprising classes of Neolithic slate plaques from the southwestern Iberian Peninsula, several character-state polarities suggested by Lillios seem broadly successful. After all, people learn from those with whom they are culturally related and/or with those with whom they are in contact, and ideas as well as people move across the landscape. Thus we should expect *some* structure in the data. In fact, given the manner in which we stacked the analysis in favor of Lillios's hypothesis, one might have expected *more* structure in the plaques from the southwestern Iberian Peninsula, irrespective of whether they served the purpose(s) assigned to them. Certainly the available stratigraphic evidence and radiocarbon dates do not impart clear chronological structure to the plaques, which is yet another strike against Lillios's hypothesis that they served as genealogical mnemonic recording systems.
